# Hydrocortisone in treatment of severe sepsis and septic shock with acute respiratory distress syndrome: a randomised controlled trial

**DOI:** 10.1186/2197-425X-3-S1-A808

**Published:** 2015-10-01

**Authors:** S Tongyoo, C Permpikul, V Vattanavanit, W Mongkolpun

**Affiliations:** Siriraj Hospital, Mahidol University, Critical Care Medicine, Bangkok, Thailand; Prince of Songkla University, Songkla, Thailand

## Introduction

Severe sepsis and septic shock are serious conditions associated with high mortality, especially in the patients complicated with acute respiratory distress syndrome (ARDS). Corticosteroids are recommended in patients who do not respond to fluid resuscitation and vasopressors. However, efficacy of this treatment in severe sepsis patients with ARDS has not been investigated.

## Objectives

To examine efficacy of moderate dose of hydrocortisone in the treatment of severe sepsis/septic shock complicated with ARDS.

## Methods

A single center, placebo-controlled, randomized, double blind study which included adult patients with severe sepsis or septic shock and ARDS, onset within 24 hours. They were randomly assigned into two groups. The “study group” received hydrocortisone 50 mg intravenous every 6 hours for 7 days, while the “controlled group” received normal saline in the comparable volume. The primary outcome was 28 days mortality.

## Results

A total of 197 patients were included. Ninety eight were in “study group” and 99 were in the “control group”. While baseline characteristics were similar, the study group had longer organ support-free days in the 28 days (13.9+10.3 vs 10.8+10.1, P = 0.04), shorter vasopressor-dependent days (3.8+3.3 vs 5.2+5.7, P = 0.04), lower lung injury score at the 3^rd^ day after treatment (1.5+0.8 vs 1.8+1.0, P = 0.03) and improve PaO_2_/FiO_2_ ratio at the 3^rd^ day after treatment (273.9+126.9 vs 227.6+110.3, P = 0.007). The incidence of hyperglycemia was also higher in the study group. Mortality in the study group was lower although statistical difference was not reached (25.5% vs 31.3%, P = 0.37).

## Conclusions

Hydrocortisone improved outcomes; namely organ support-free days, vasopressor-dependent days and oxygenation, in severe sepsis or septic shock patients with ARDS. There was a trend toward mortality reduction in the study group but the statistical significant was not reached. (ClinicalTrials.gov number, NCT01284452)

## Grant Acknowledgment

Facualty of Medicine, Siriraj Hospital, Mahidol University.
